# High-level expression of the HIV entry inhibitor griffithsin from the plastid genome and retention of biological activity in dried tobacco leaves

**DOI:** 10.1007/s11103-018-0744-7

**Published:** 2018-06-09

**Authors:** Matthijs Hoelscher, Nadine Tiller, Audrey Y.-H. Teh, Guo-Zhang Wu, Julian K-C. Ma, Ralph Bock

**Affiliations:** 1 Max-Planck-Institut für Molekulare Pflanzenphysiologie, Am Mühlenberg 1, 14476 Potsdam-Golm, Germany; 2 Institute for Infection and Immunity, St. George’s, University of London, Cranmer Terrace, London, SW17 0RE UK; 3 Present Address: Leibniz Universität Hannover, Herrenhäuser Straße 2, 30419 Hannover, Germany

**Keywords:** Plastid transformation, *Nicotiana tabacum*, Molecular farming, HIV, AIDS, Antiviral agent, Microbicide, Griffithsin, Chloroplast

## Abstract

**Key message:**

The potent anti-HIV microbicide griffithsin was expressed to high levels in tobacco chloroplasts, enabling efficient purification from both fresh and dried biomass, thus providing storable material for inexpensive production and scale-up on demand.

**Abstract:**

The global HIV epidemic continues to grow, with 1.8 million new infections occurring per year. In the absence of a cure and an AIDS vaccine, there is a pressing need to prevent new infections in order to curb the disease. Topical microbicides that block viral entry into human cells can potentially prevent HIV infection. The antiviral lectin griffithsin has been identified as a highly potent inhibitor of HIV entry into human cells. Here we have explored the possibility to use transplastomic plants as an inexpensive production platform for griffithsin. We show that griffithsin accumulates in stably transformed tobacco chloroplasts to up to 5% of the total soluble protein of the plant. Griffithsin can be easily purified from leaf material and shows similarly high virus neutralization activity as griffithsin protein recombinantly expressed in bacteria. We also show that dried tobacco provides a storable source material for griffithsin purification, thus enabling quick scale-up of production on demand.

**Electronic supplementary material:**

The online version of this article (10.1007/s11103-018-0744-7) contains supplementary material, which is available to authorized users.

## Introduction

The global human immunodeficiency virus (HIV) epidemic continues to grow, with more than 36.7 million people now estimated to carry the virus (UNAIDS Report 2017: http://www.unaids.org/en/resources/documents/2017/2017_data_book). Although global scale-up of antiretroviral therapy has recently resulted in a substantial decline in deaths from AIDS-related causes, HIV was still responsible for approximately a million deaths in 2016. At the same time, 1.8 million new infections occur per year, resulting in a continuous net increase in infected individuals (UNAIDS Report 2017: http://www.unaids.org/en/resources/documents/2017/2017_data_book). In the absence of a cure that would eliminate the virus, it is therefore of utmost importance to reduce the number of new infections. Unfortunately, despite enormous efforts to develop immunization strategies against HIV, an AIDS vaccine is currently not in sight (Levine 2008; Virgin and Walker 2010; Haase 2010; Andrabi et al. 2018). Topical (vaginal and rectal) microbicides that potentially prevent new infections, therefore, represent one of the most promising strategies to control the disease (Boyd et al. 1997; Lederman et al. 2004; Mori et al. 2005; Veazey et al. 2005; Rao et al. 2005; Vanpouille et al. 2012; O’Keefe et al. 2015).

Inhibitors that block entry of the virus into human cells are particularly suitable to prevent infection (Lederman et al. 2004; Veazey et al. 2005). Large-scale screens for peptides, proteins and small molecules that act as virus-cell fusion inhibitors have been conducted and a number of candidate molecules have been identified. One of the first potent antiviral agents that was found to bind to the HIV surface and block viral entry into cells was cyanovirin-N (CV-N), a small protein (11 kDa) of the cyanobacterium *Nostoc ellipsosporum* (Boyd et al. 1997). It binds irreversibly to the surface envelope glycoprotein gp120 by targeting N-linked high-mannose oligosaccharides (Botos et al. 2002; Bewley et al. 2002; Barrientos and Gronenborn 2002) and, in this way, blocks binding of the virus to its receptors on host cells. Recombinant production of CV-N has been attempted in several systems, including bacteria, transgenic plants and transplastomic plants, but protein yields have been relatively low (Colleluori et al. 2005; Sexton et al. 2006; Gao et al. 2010; Elghabi et al. 2011; O’Keefe et al. 2015).

An even more potent small protein with a similar mode of action is the 12.7 kDa lectin griffithsin from the red alga *Griffithsia* sp. (Mori et al. 2005; Kouokam et al. 2011). While CV-N is active against HIV at low nanomolar concentrations, griffithsin displays anti-viral activity against all isolates of HIV already at picomolar concentrations (Mori et al. 2005; O’Keefe et al. 2009), thus making griffithsin a highly attractive candidate microbicide to prevent sexual transmission of the virus. In addition to its high specific activity against HIV, griffithsin is also extremely resistant to physicochemical degradation and shows high safety (Kouokam et al. 2011). Preclinical development of griffithsin as a potential vaginal microbicide for prevention of HIV transmission is currently underway. Interestingly, griffithsin also displays strong affinity to the envelope glycoproteins of other highly pathogenic viruses, such as the SARS coronavirus (SARS-CoV), hepatitis C virus (HCV) and Ebola virus (O’Keefe et al. 2010; Barton et al. 2014).

Development of griffithsin as an affordable anti-HIV microbicide will require inexpensive mass production of the protein, ideally from a renewable source and not requiring expensive fermentation procedures. Plants represent a particularly cheap, readily scalable and safe production platform for biopharmaceuticals (Ma et al. 2005; Daniell et al. 2009; Rybicki 2010; Marusic et al. 2009; Bock 2014, 2015; Wong-Arce et al. 2017). Therefore, extensive efforts have been made to develop technologies that enable high-level expression of recombinant proteins in plants, including antibodies, antigens for subunit vaccines and microbicides. Expression of griffithsin has been tested in several expression systems, including viral vector-based transient expression in tobacco leaves (O’Keefe et al. 2009; Hahn et al. 2015; Fuqua et al. 2015a, b) and stable transgenic expression in rice seeds (Vamvaka et al. 2016). While transient expression systems often give high expression levels, they incur additional costs due to the need to transfect each new batch of plant material. By contrast, in stable transgenic plants, the starting material for purification can be provided at the (very low) cost of the biomass. Also, stable transgenic plants provide greater batch-to-batch consistency than transiently transfected plant material (Fujiuchi et al. 2016).

Transgene expression from the plastid (chloroplast) genome offers a number of highly attractive features, including precise transgene insertion by homologous recombination, absence of epigenetic gene silencing, and greatly increased transgene confinement due to maternal inheritance of plastids and their efficient exclusion from pollen in most crops (Maliga 2004; Ruf et al. 2007; Bock 2015). The greatest attraction for molecular farming applications lies in the potential of chloroplast-transformed (transplastomic) plants to accumulate extraordinarily high levels of foreign proteins, often one to three orders of magnitude higher than what is possible to achieve by nuclear transgenesis (De Cosa et al. 2001; Tregoning et al. 2003; Molina et al. 2004; Zhou et al. 2008; Oey et al. 2009a, b). However, it is important to note that, while there are numerous cases of spectacularly high expression levels achieved in transplastomic plants, not all foreign proteins accumulate stably in transgenic chloroplasts. Unfortunately, the rules governing protein stability in plastids are only poorly understood (Apel et al. 2010; De Marchis et al. 2012) and, thus, predictions about the success of recombinant protein accumulation in chloroplasts are currently hardly possible. For example, transplastomic expression of CV-N in tobacco was undetectably low, but could be improved by fusion to N-terminal and C-terminal sequences taken from proteins known to be very stable in plastids (Elghabi et al. 2011). Nonetheless, CV-N accumulation levels reached only 0.3% of the total soluble protein (TSP), a value that is two orders of magnitude below the levels attained with PlyGBS, one of the proteins whose N- and C-terminal sequences were used for stabilization of CV-N (Oey et al. 2009a; Elghabi et al. 2011).

Here we have explored the possibility to express griffithsin in tobacco chloroplasts. Although CV-N and griffithsin are similar in size and have a similar mode of antiviral action, their primary sequences are very different. Also, CV-N is a prokaryotic (cyanobacterial) protein, while griffithsin is of eukaryotic (red algal) origin. Thus, the two proteins likely evolved different (in)stability determinants which may have different effects on their stability in the chloroplast (representing a prokaryotic system). Expression of griffithsin from the chloroplast genome was attempted recently (Vafaee et al. 2014), but no accumulation levels were reported and no purification and activity tests were performed. We report here that griffithsin can be expressed to very high levels (of approximately 5% of the plant’s total soluble protein) in tobacco chloroplasts and has similar anti-HIV activity as the recombinant protein produced in bacteria. We also show that, due to its enormous thermal stability, (i) griffithsin can be very easily purified from leaf biomass, (ii) high levels of intact protein can be extracted from dried leaves, and (iii) griffithsin isolated from dry tobacco retains high antiviral activity.

## Materials and methods

### Plant material and growth conditions

Sterile tobacco (*Nicotiana tabacum* cv. Petit Havana) plants were grown on agar-solidified MS medium (Murashige and Skoog 1962) containing 30 g/L sucrose. For seed production and generation of material for molecular analyses, plants were transferred to soil and cultivated under standard greenhouse conditions. For inheritance assays, seeds were surface sterilized and sown on agar-solidified MS medium containing 20 g/L sucrose and the appropriate antibiotics for selection (spectinomycin: 500 mg/L, gentamycin: 200 mg/L).

To investigate the recovery of griffithsin from dry tobacco, leaves were harvested from greenhouse-grown plants, dried between tissue paper for approximately 4 months at room temperature, followed by initial tests for the presence of griffithsin and subsequent storage in sealed containers at room temperature until 10 months after harvest, followed by protein purification.

### Cloning procedures

The griffithsin nucleotide sequence (accession number AY744144; Mori et al. 2005) including the sequence for an N-terminal histidine tag was codon-optimized for the tobacco chloroplast genome and chemically synthesized (Epoch Life Science, Missouri City, TX; http://www.epochlifescience.com/). The synthetic *grft* gene was inserted as NdeI/XbaI fragment into cloning vector pHK20 (Kuroda and Maliga 2001) where it is driven by the plastid rRNA operon promoter fused to the *gene10* leader sequence of bacteriophage T7. The griffithsin cassette was subsequently excised as SacI/HindIII fragment and cloned into the similarly cut plasmid pKP9 (Zhou et al. 2008), generating the final transformation vector pGrft1.

### Chloroplast transformation

Young tobacco leaves were bombarded with vector pGrft1 coated onto gold particles (0.6 µm diameter) using a helium-driven biolistic gun (PDS-1000He; Bio-Rad). Primary transformants were selected on regeneration medium containing 500 mg/L spectinomycin (Svab and Maliga 1993) and subsequently subjected to two additional rounds of regeneration on spectinomycin-containing medium to select against residual copies of the wild-type plastid genome. Spontaneous spectinomycin-resistant lines were eliminated by double selection on medium containing both spectinomycin and streptomycin (500 mg/L each; Svab and Maliga 1993; Bock 2001). Homoplasmic transplastomic plantlets were rooted on hormone-free medium and then transferred to the greenhouse for seed production.

### Selectable marker gene removal

For marker elimination, homoplasmic transplastomic plants were crossed to nuclear-transgenic plants expressing a plastid-targeted Cre recombinase (Corneille et al. 2001). The resulting seeds were surface sterilized and cultivated on MS medium containing 20 g/L sucrose and 500 mg/L spectinomycin. White seedlings were transferred to antibiotic-free medium and, after greening and continued growth, transferred to the greenhouse. To also eliminate the nuclear marker gene, plants were pollinated with wild-type pollen. The progeny from these crosses was used for protein purification experiments.

### Isolation of nucleic acids and gel blot analyses

Total plant DNA was extracted by a cetyltrimethylammoniumbromide-based method (Doyle and Doyle 1990). Total cellular RNA was isolated with the NucleoSpin RNA Plant kit (Macherey–Nagel). For RFLP analysis, DNA samples (3 µg total DNA) were treated with the restriction enzyme BamHI, followed by separation of the fragments by electrophoresis in 1% agarose gels and blotting onto Hybond XL membranes (GE Healthcare). For northern blot analysis, RNA samples were electrophoretically separated in formaldehyde-containing 1.5% agarose gels and transferred onto Hybond XL membranes.

A restriction fragment covering the entire coding region of the selectable marker gene *aadA* was purified by agarose gel electrophoresis using the NucleoSpin Extract II kit (Macherey–Nagel) and used as a hybridization probe. Hybridization probes against *psaB* and *grft* were generated by PCR using gene-specific primers (PpsaB_for 5′-CCCAGAAAGAGGCTGGCCC-3′ and PpsaB_rev 5′-CCCAAGGGGCGGGAACTGC-3′; Pgrft_for 5′-ATGGGATCTTCTCATCATCATC-3′ and Pgrft_rev 5′-TTAATATTGTTCATAATAAATATCTAAAGA-3′). The resulting amplification products of 550 bp and 420 bp, respectively, were purified and probes were labelled with α[^32^P]dCTP by random priming (GE Healthcare). Hybridizations were performed at 65 °C in Rapid-Hyb buffer (GE Healthcare) according to the manufacturer’s protocol.

### Protein isolation, purification and visualization

Total soluble protein was extracted from leaf samples homogenized in a buffer (buffer 1) containing 50 mM *N*-2-hydroxyethylpiperazin-*N′*-2-ethanesulfonic acid (HEPES)-KOH (pH 7.5), 10 mM potassium acetate, 5 mM magnesium acetate, 1 mM EDTA, 1 mM dithiothreitol and 1 mM cOmplete protease inhibitor (Roche). Alternatively, a published extraction buffer (buffer 2: 100 mM Tris, 300 mM NaCl, 20 mM ascorbic acid, 10 mM sodium metabisulfite; Fuqua et al. 2015a, b) was used. Total soluble protein concentrations were determined by the Bradford assay (Roche, Karlsruhe, Germany) using known concentrations of bovine serum albumin (BSA) as a protein standard.

Extraction and purification protocols for griffithsin from tobacco leaves were optimized for yield and purity. Different extraction buffers were tested: (1) the standard native His-tag purification buffers described by QIAGEN (50 mM NaH_2_PO_4_, 300 mM NaCl, cOmplete protease inhibitor with or without 1% Triton, including 10 mM imidazole for extraction, 20 mM imidazole for washing and 250 mM imidazole for elution; pH 8 with NaOH), and (2) previously published extraction buffers for griffithsin (Fuqua et al. 2015a, b; 100 mM sodium acetate, 300 mM sodium chloride, 20 mM ascorbic acid, 10 mM sodium metabisulfite, with or without 100 mM MgCl_2_, pH 4 or 6 with HCl; or 100 mM Tris, 300 mM sodium chloride, 20 mM ascorbic acid, 10 mM sodium metabisulfite, with or without 100 mM MgCl_2_, pH 8 with HCl). When used for His-tag-based purification, the buffers were modified by inclusion of 10 mM imidazole for extraction. The pH 8 buffer was modified by including 20 mM imidazole for washing and 250 mM imidazole for elution.

For extraction, leaves were frozen in liquid nitrogen, ground and suspended in 200 µL of extraction buffer per 100 mg of material. For dried leaves, 2900 µL buffer were added per 100 mg material. The suspension was kept on ice and vortexed every 5 min, followed by centrifugation at 15,000×*g* for a total of 15 min at 4 °C. Alternatively, the suspension was incubated under shaking (Eppendorf ThermoMixer comfort) at 700 or 1400 rpm for 15 min at 70 °C followed by centrifugation at 15,000×*g* for 15 min at 4 °C, 16 °C or 22 °C, or the suspension was centrifuged for 15 min and the supernatant heated for 15 min at 70 °C under shaking at 700 or 1400 rpm, followed by a second centrifugation step of 15 min at 15,000×*g* at 4 °C. The supernatant from the heat-treated samples was used for His-tag-based protein purification via Ni–NTA Agarose (QIAGEN) following the manufacturer’s protocol. 3 mL of Ni–NTA agarose slurry were used for purification from approximately 8 g of fresh tissue or 1 g of dried tissue (i.e., approximately 25–30 mL of leaf extract). Plant extracts in pH 4 buffer were adjusted to pH 8 with NaOH prior to mixing with Ni–NTA agarose. The Ni–NTA agarose was incubated with the leaf extract for 1 h (rotating at 4 °C) and subsequently loaded onto QIAGEN Polypropylene Columns (5 mL). The eluate from the purification was concentrated and dialyzed into PBS buffer (137 mM NaCl, 2.7 mM KCl, 10 mM Na_2_HPO_4_, pH 7.4) by ultrafiltration with Amicon ® Ultra-4 centrifugal filters (Merck) with a 3000 NMWL cut-off, reducing the sample to approximately one-tenth of the original volume. The concentration of ultrafiltrated griffithsin samples was determined with the Pierce BCA Protein Assay Kit (Thermo Fisher Scientific) using a dilution standard of BSA and recombinant 6xHis-tagged griffithsin (purified from *E. coli*; NIH AIDS Research & Reference Reagent Program, Germantown, MD) as reference proteins.

To visualize accumulation, extraction efficiency and purity, protein extracts were electrophoretically separated in 10% Tris-Tricine/SDS polyacrylamide gels (Schägger and von Jagow 1987), and either directly visualized by Coomassie blue staining or silver staining, or transferred to Hybond-P PVDF membranes (GE Healthcare) with transfer buffer (192 mM glycine, 25 mM Tris, pH 8.3) in a Trans-Blot cell (Bio-Rad) for 2 h at 1 A. Blots were blocked with 3% BSA and immunodetection was performed with a griffithsin-specific antibody (NIH AIDS Research & Reference Reagent Program) and goat anti-rabbit IgG antibody peroxidase conjugate (Agrisera) as secondary antibody. Detection was performed with an enhanced chemiluminescence system (ECL® PLUS; GE Healthcare). Recombinant histidine-tagged griffithsin from *E. coli* was used as reference.

### Determination of anti-HIV activity of purified griffithsin

HIV-1 BaL neutralization assays were performed according to published procedures (Wei et al. 2003; Montefiori 2005). 200 TCID50 (median tissue culture infective dose) of HIV-1 BaL were incubated with dilutions of purified griffithsin produced in tobacco or *E. coli* for 1 h at 37 °C in a total volume of 100 µL. Tobacco- and *E. coli-*produced griffithsin were titrated fivefold seven times in triplicate starting at 2 µg/mL. All dilutions were performed in growth medium (DMEM) supplemented with 10% foetal calf serum (FCS), penicillin–streptomycin (100 units/mL and 100 µg/mL, respectively) and 2 mM L-glutamine. The mixture was then added to 100 µL of TZM-bl cells expressing CD4, CCR5 and the firefly luciferase gene under the control of the HIV long-terminal repeat sequence and grown in medium pre-seeded at a concentration of 1 × 10^5^ cells/mL. A positive control (cells plus virus) and a cell-only background control were also included. After a 24 h incubation at 37 °C in 5% CO_2_ followed by addition and incubation with lysis buffer, the cell lysate was removed and mixed in a 1:1 ratio with Bright Glo reagent (Promega) in a 96-well black solid plate for luminescence measurements. The percentage reduction in relative light units (RLU) was calculated relative to the RLU of the positive control (cells plus virus). The resulting curve was plotted and analyzed in GraphPad Prism (https://www.graphpad.com/).

## Results

### Stable transformation of the tobacco plastid genome with a griffithsin expression cassette

**Fig. 1:**
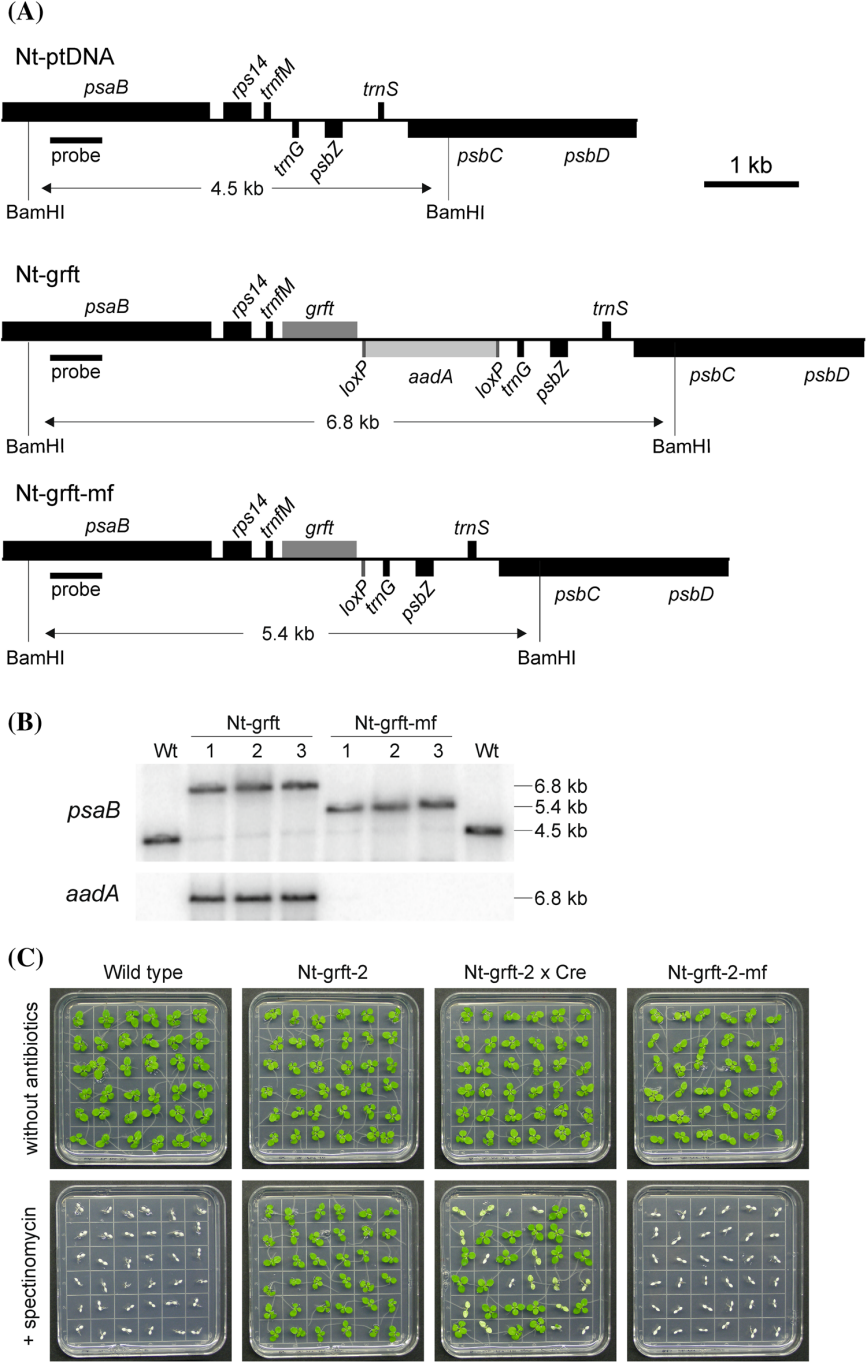
Generation of transplastomic tobacco plants expressing the anti-HIV protein griffithsin. **a** Physical maps of the targeted region in the tobacco plastid genome (Nt-ptDNA) and the transgenic plastid genomes in Nt-grft and Nt-grft-mf lines. The transgenes are targeted to the intergenic region between two plastid tRNA genes, *trnfM* and *trnG* (Zhou et al. 2008). Expression of the *grft* transgene is driven by the plastid rRNA operon promoter fused to the *gene10* leader sequence of coliphage T7 and the 3′ UTR of the plastid *rbcL* gene. The *aadA* expression cassette consists of a chimeric plastid rRNA operon promoter and the 3′ UTR of the *psbA* gene (Svab and Maliga 1993). The expected sizes of the DNA fragments in RFLP analyses with the restriction enzyme BamHI are given below each map. The location of the hybridization probe is indicated as a black bar. **b** RFLP analysis of transplastomic Nt-grft and Nt-grft-mf plants. DNA samples were digested with BamHI and hybridized to a radiolabelled probe detecting the plastid genome region flanking the transgene insertion site. The homoplasmic state was evidenced by absence of the 4.5 kb fragment characteristic of the wild-type plastid genome and detection of the 6.8 kb fragment specific to the transformed plastid genome. Successful post-transformation excision of the *aadA* gene (Zhou et al. 2008) and generation of marker-free griffithsin-expressing transplastomic plants (Nt-grft-mf) was initially assayed with the *psaB* probe (detecting a 5.4 kb fragment) and further confirmed by hybridization to an *aadA*-specific probe (which exclusively detected the expected 6.8 kb fragment in the marker gene-containing Nt-grft plants).* Wt* wild type. **c** Inheritance assay and identification of marker-free transplastomic lines. Germination of the T1 generation of Nt-grft plastid transformants in the presence of spectinomycin revealed homoplasmic presence of the selectable marker gene *aadA*. While wild-type seedlings emerge white in the presence of the antibiotic, all seedlings of transplastomic plants are uniformly resistant to spectinomycin. The progeny of Nt-grft plants crossed to nuclear-transgenic plants expressing a plastid-targeted Cre recombinase (Nt-grft-2 × Cre) segregates into 50% fully green seedlings (due to hemizygous presence of the *cre* recombinase gene in the parental line), variegated and completely white seedlings in the presence of spectinomycin. The white seedlings, expected to be completely devoid of the *aadA* marker, were rescued by transfer to antibiotic-free medium, subsequently grown to maturity in the greenhouse and pollinated with wild-type pollen. Uniformly white appearance of the progeny (Nt-grft-mf) upon germination on spectinomycin-containing medium confirmed the complete removal of the *aadA* marker

To test whether the anti-HIV protein griffithsin can be expressed from the chloroplast genome to high levels, the sequence of the griffithsin gene (*grft*) was codon optimized by adjusting it to the preferred codon usage in the tobacco plastid DNA (Shimada and Sugiura 1991). To facilitate protein purification, an N-terminal histidine tag (His_6_) was added to the coding region (NCBI accession number AY744144.1). The synthetic gene construct was inserted into an expression cassette consisting of the ribosomal RNA operon promoter (P*rrn*) fused to the strong translation initiation signals derived from the *gene10* leader of coliphage T7 (Kuroda and Maliga 2001; see Materials and Methods). The griffithsin cassette was then integrated into a standard plastid transformation vector (Zhou et al. 2008) containing a chimeric spectinomycin resistance gene (*aadA*) as selectable marker. In this vector, the *aadA* gene is flanked by *loxP* sites to facilitate post-transformation removal of the antibiotic resistance marker (Fig. 1a; Corneille et al. 2001).

Biolistic plastid transformation experiments in tobacco were followed by selection for resistance to spectinomycin (Svab and Maliga 1993). Primary spectinomycin-resistant clones (Nt-grft lines) were subjected to additional rounds of selection and regeneration to dilute out residual wild-type copies of the highly polyploid plastid genome and isolate homoplasmic transplastomic tissue. Successful transformation and correct targeting of the *grft* and *aadA* transgenes to the intergenic spacer separating the *trnfM* and *trnG* genes by homologous recombination was analyzed by restriction fragment length polymorphism (RFLP) analysis using Southern blotting (Fig. 1a, b). The results showed the expected hybridization patterns in RFLP analyses with either a probe derived from a neighboring gene in the plastid genome or a probe recognizing the *aadA* marker gene (Fig. 1b). Absence of a hybridization signal for the wild type-specific fragment preliminarily suggested that the lines were homoplasmic. However, strong exposure of the blots revealed faint hybridization signals corresponding to the expected size of the wild-type plastid DNA fragment (Fig. 1b, and data not shown). Previous work had established that these weak signals come from so-called promiscuous DNA (plastid-derived DNA sequences that are present in the nuclear genome; Hager et al. 1999; Ruf et al. 2000).

To ultimately confirm homoplasmy of the Nt-grft lines, we conducted large-scale inheritance tests by germinating T1 seeds on spectinomycin-containing medium (Bock 2001). No appearance of antibiotic-sensitive seedlings was observed in any of the analyzed lines (Fig. 1c, and data not shown), indicating that the transplastomic Nt-grft lines are homoplasmic.

### Selectable marker gene removal from transplastomic plants

To increase the public acceptance of the use of transgenic plants in biotechnology, the post-transformation removal of antibiotic resistance markers is highly desirable. Excision of the *aadA* cassette is also useful to prevent rearrangements or deletions in the transformed genome due to unwanted homologous recombination between the expression elements controlling the *aadA* cassette (promoter, 5′ UTR and 3′UTR sequences) and the resident copies of these expression elements in the plastid genome (Rogalski et al. 2006).

**Fig. 2:**
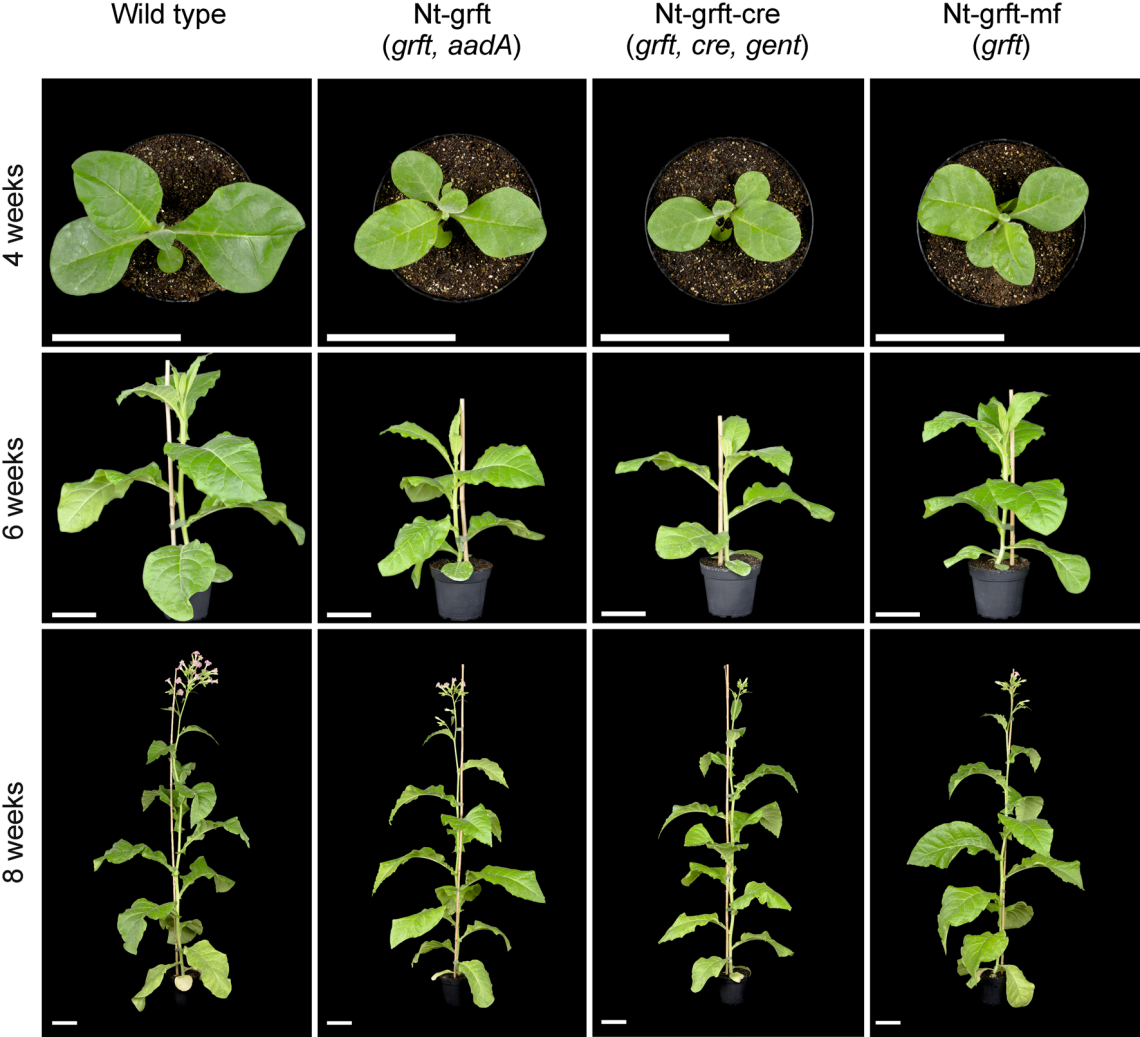
Plant phenotypes upon growth in soil. Plants were raised from seeds and photographed 4 weeks (29 days), 6 weeks and 8 weeks after germination. A wild-type plant, a transplastomic plant harboring the *grft* and *aadA* transgenes (Nt-grft line), a transplastomic plant lacking the *aadA* marker due to excision by a nucleus-encoded chloroplast-targeted Cre recombinase (also carrying the linked nuclear gentamycin resistance marker *gent*; Nt-grft-cre) and transplastomic *grft* plants without any other transgenes (Nt-grft-mf) are shown. The transgenes present in each line are given in parentheses. Scale bars: 10 cm

Use of a *loxP*-flanked (‘floxed’) version of the *aadA* marker gene in our transformation construct (Fig. 1a) allowed excision of the *aadA* cassette by site-specific recombination triggered by a nucleus-encoded Cre recombinase that is post-translationally imported into plastids (Corneille et al. 2001). To this end, we crossed homoplasmic transplastomic Nt-grft lines (maternal parent) to a nuclear transgenic Cre-expressing line (pollen donor). The progeny was raised on spectinomycin-containing medium to identify seedlings whose plastid genome was devoid of the *aadA* gene due to Cre-mediated excision from all copies of the plastid genome. These seedlings were easily recognizable by their spectinomycin-sensitive (pale) phenotype. As expected, the progeny segregated into homogeneously pale (complete *aadA* excision), variegated (incomplete *aadA* excision) and green (no or low-level *aadA* excision) seedlings (Fig. 1c). Homogeneously pale seedlings were rescued by transfer to antibiotic-free medium, after recovery grown to maturity in the greenhouse and then crossed to the wild type (as pollen donor) to eliminate the *Cre* transgene and the linked nuclear antibiotic resistance marker (a gentamycin resistance gene; Corneille et al. 2001) in the next generation. The resulting completely marker-free transplastomic plants (Nt-grft-mf) have no nuclear transgene anymore, lack the *aadA* marker in the plastid genome and contain *grft* as the sole transgene (Figs. 1c, 2).

RFLP analyses by Southern blotting and inheritance assays ultimately confirmed that the *aadA* gene had been completely excised from all copies of the transgenic plastid genome (Fig. 1b, c).

Neither the Nt-grft lines nor the derived marker-free Nt-grft-mf lines displayed any obvious phenotypic abnormalities. Upon transfer to soil and growth under standard greenhouse conditions, all transplastomic plants were indistinguishable from wild-type plants (Fig. 2), with the exception of a slightly delayed initial development (Fig. S1a). However, this was a very subtle phenotype that became less obvious with time and had largely disappeared after a few weeks of growth (Fig. 2).

### Griffithsin mRNA and protein accumulation in transplastomic plants

**Fig. 3:**
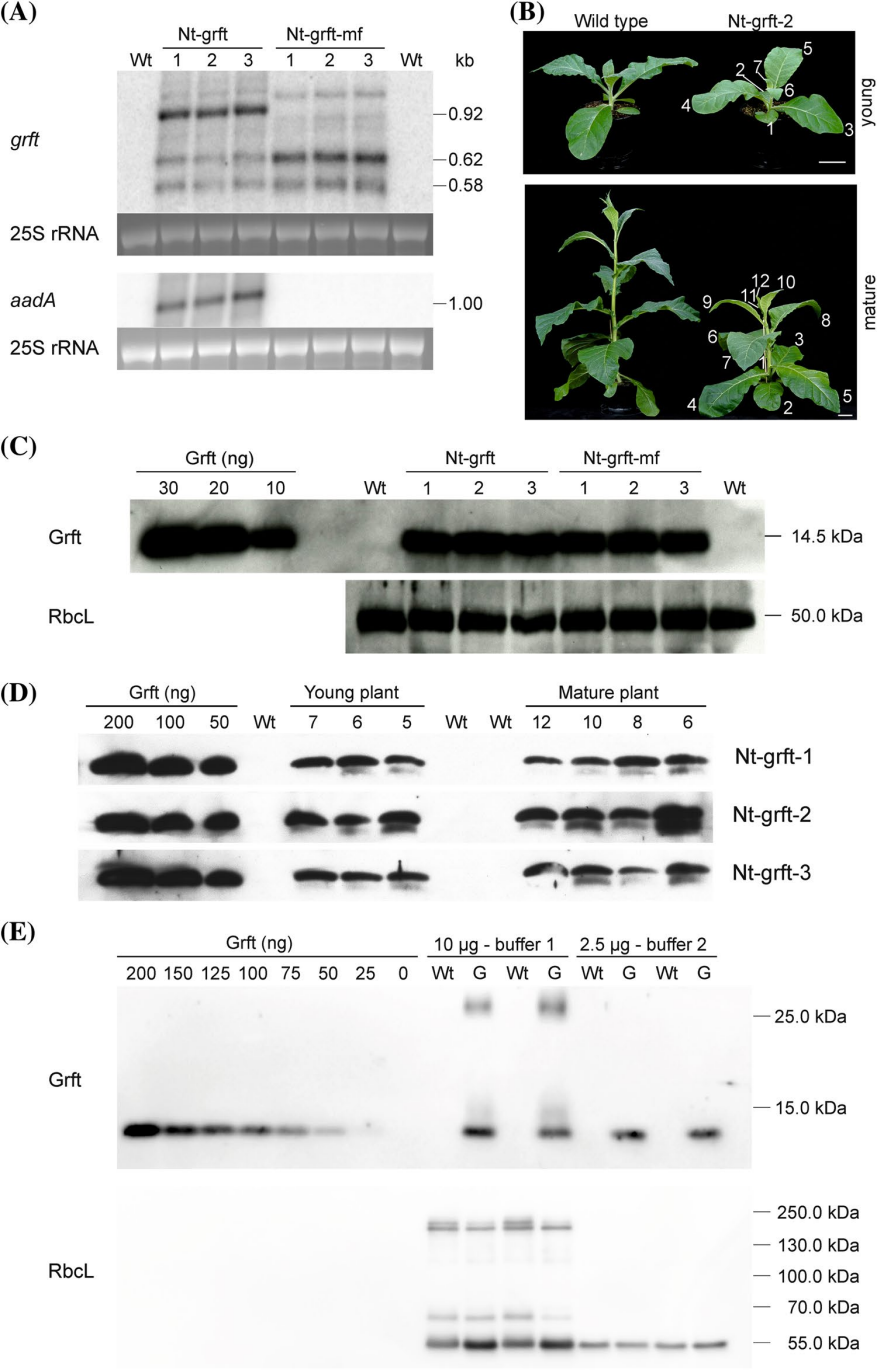
Expression of griffithsin in transplastomic tobacco plants. **a** Accumulation of *grft* and *aadA* transcripts. The *grft*-specific probe detects three major transcript species in Nt-grft plants (~ 0.92, ~ 0.62, ~ 0.58 kb) and two major transcripts in Nt-grft-mf plants (~ 0.62, ~ 0.58 kb), a pattern observed for the pKP9 expression cassette in previous studies (e.g., Zhou et al. 2008). The ~ 1 kb *aadA* transcript accumulates in Nt-grft plants, but is not detectable in the wild type (Wt) and in marker gene-free plants (Nt-grft-mf). The ethidium bromide-stained 25S rRNA of the cytosolic 80S ribosomes served as a loading control. **b** A transplastomic plant (Nt-grft-2) and a wild type grown under standard greenhouse conditions for six (young) and eight (mature) weeks to illustrate the sampling of leaves (consecutively numbered from the bottom to the top of the plant) for immunoblot analyses. Scale bar: 5 cm. **c** Accumulation of the griffithsin (Grft) protein in seedlings. The griffithsin-specific antibody detects a protein of the expected size (14.5 kDa) in total soluble protein extracts (5 µg loaded per lane) of all transplastomic plants. Immunodetection of RbcL served as loading control. **d** Comparison of griffithsin accumulation in a developmental series of leaves. Immunodetection of griffithsin in total soluble protein extracts (5 µg per sample) of three different leaves from a young plant (leaves number 7, 6 and 5) and four leaves from a mature plant (leaves 12, 10, 8 and 6). **e** Determination of griffithsin accumulation levels in total soluble protein (TSP) samples. TSP was extracted with two different buffers (see “Materials and methods”) from the wild type (Wt) and the Nt-grft-mf-2 line (G). Two replicates per buffer and plant line were analyses. For buffer 1, samples of 10 µg TSP and for buffer 2, samples of 2.5 µg TSP were loaded. Immunodetection of RbcL served as loading control. In buffer 1, the griffithsin dimer is detected and RbcL shows larger complexes, suggesting incomplete protein denaturation. Using known standards of recombinant histidine-tagged griffithsin, griffithsin was detected at approximately 2% of TSP with buffer 1 (including the dimer) and approximately 5% with buffer 2

To test for expression of the introduced *grft* transgene, we first performed northern blot experiments using transgene-specific probes. Hybridization to a *grft* probe detected a band of the expected size of the mature (monocistronic) *grft* mRNA (0.58 kb; Fig. 3a) and two larger transcript species that likely result from read-through transcription and termination or processing at the *loxP* site downstream of the *grft* gene, as observed previously with transgenes expressed from the same insertion site in the plastid genome (e.g., Zhou et al. 2008). The Nt-grft-mf lines lack a prominent band for the largest transcript species seen in the Nt-grft plants, tentatively suggesting that this RNA species originates from termination or processing within the *aadA* cassette downstream of the *grft* gene (Figs. 1a, 3a). As expected, the Nt-grft-mf lines lack *aadA* transcripts (Fig. 3a, bottom panel), confirming complete marker gene removal from all copies of the plastid genome.

Having confirmed stable *grft* mRNA accumulation, we next wanted to determine accumulation of the griffithsin protein in chloroplasts. To this end, we conducted a series of western blot analyses using anti-griffithsin antibodies and purified His-tagged griffithsin protein recombinantly expressed in *E. coli* as a standard for protein quantification (Fig. 3b–e). Since foreign protein accumulation in transplastomic plants can be strongly dependent on leaf age (e.g., Zhou et al. 2008; Oey et al. 2009a, b), we also analyzed a developmental series of leaves of different ages harvested from young or mature transplastomic plants (Fig. 3b).

Western blot analysis revealed a strong signal for the recombinant His-tagged griffithsin protein at the expected size of ~ 14.5 kDa in all transplastomic plants (Fig. 3c). Comparison of Nt-grft with Nt-grft-mf lines revealed no significant differences, indicating that marker excision does not impact recombinant protein accumulation (Fig. 3c). Analysis of Nt-grft leaves of different ages and developmental stages also did not show pronounced differences in griffithsin accumulation levels (Fig. 3d). This finding was further confirmed by Coomassie staining of protein extracts from Nt-grft-mf lines which revealed a strong band for griffithsin in leaves of all ages and developmental stages, with the exception of the very young leaflets (close to the apical meristem) that accumulated substantially lower levels of griffithsin protein (Fig. S1).

Quantification of griffithsin accumulation using two different buffers for extraction of total soluble protein (TSP) yielded slightly different values, presumably because one of the buffers (buffer 1) was less efficient in extraction of griffithsin from leaf material, as also suggested by incomplete denaturation of the griffithsin dimers into monomers (Fig. 3e). Following extraction with the more effective buffer 2, griffithsin was detected at approximately 5% of TSP (Fig. 3e), which represents an extraordinarily high accumulation level, given the small size of the protein.

### Purification of griffithsin from transplastomic tobacco plants

Having achieved high levels of griffithsin accumulation in transplastomic plants, we next attempted purification of the protein to (i) be able to determine its biological activity as antiviral agent, and (ii) test if transplastomic plants provide a suitable production platform for the large-scale synthesis and isolation of pure griffithsin as biopharmaceutical. Due to its physicochemical properties and, in particular, its exceptionally high thermal stability, griffithsin can be enriched by heating total protein extracts which results in the denaturation and precipitation of most other cellular proteins (Fuqua et al. 2015a, b).

**Fig. 4:**
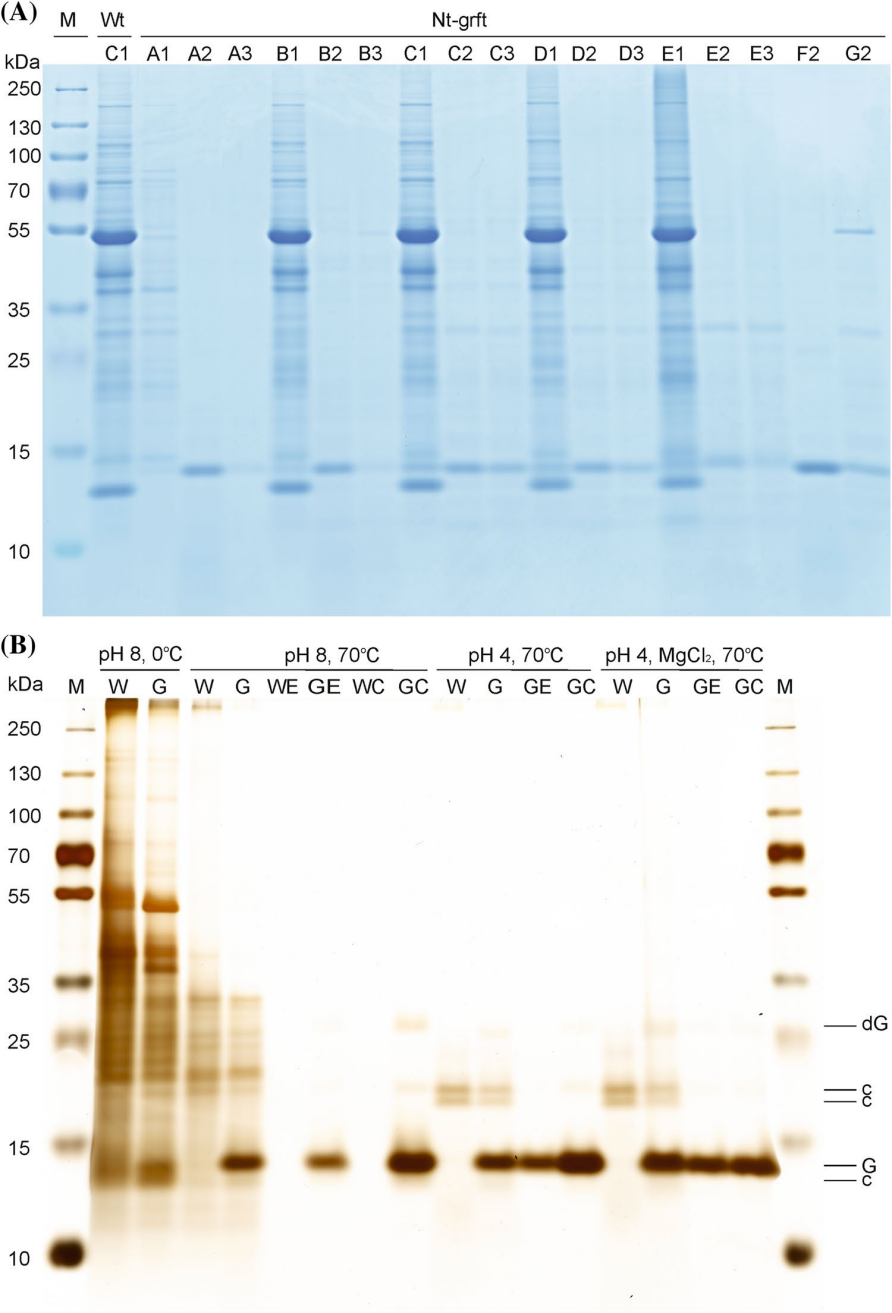
Comparison of seven different extraction buffers and three handling methods for griffithsin extraction, purification and concentration. **a** Coomassie blue-stained Tris-Tricine SDS polyacrylamide gel (10%). Buffer A: 100 mM sodium acetate, 300 mM NaCl, 20 mM ascorbic acid, 10 mM sodium metabisulfite, pH 4 (Fuqua et al. 2015a, b). Buffer B: 100 mM sodium acetate, 300 mM NaCl, 20 mM ascorbic acid, 10 mM sodium metabisulfite, pH 6 (Fuqua et al. 2015a, b). Buffer C: 100 mM Tris, 300 mM NaCl, 20 mM ascorbic acid, 10 mM sodium metabisulfite, pH 8 (Fuqua et al. 2015a, b). Buffer D: 50 mM NaH_2_PO_4,_ 300 mM NaCl, 10 mM imidazole, pH 8 (QIAGEN, 6xHis-tag purification buffer). Buffer E: buffer D with 1% Triton X-100. Buffer F: Buffer A with 0.1 M MgCl_2_. Buffer G: Buffer C with 0.1 M MgCl_2_. 1× complete EDTA-free protease inhibitor (Roche) was included in all buffers. Handling method 1: ground leaf material was incubated for 15 min in the extraction buffer on ice with vortexing every 5 min, followed by centrifugation (15 min, 15,000×*g*, 4 °C). Handling method 2: Ground leaf material was heated in the extraction buffer at 70 °C for 15 min under shaking at 1400 rpm, followed by centrifugation (15,000×*g*, 4 °C). Handling method 3: ground leaf material in extraction buffer was centrifuged at 15,000×*g* at 4 °C, followed by incubation of the supernatant at 70 °C for 15 min under shaking at 1400 rpm, followed by a second centrifugation step at 15,000×*g* at 4 °C. *Wt* wild type. **b** Silver-stained SDS polyacrylamide gel (10%) illustrating different purification methods. Extraction was performed with wild-type plants (W) and griffithsin-producing transplastomic plants (G) to test the effect of different buffers in combination with heating on the abundance of contaminants. E in the sample designation indicates eluate (from His-tag-based purification), C indicates the concentrated sample (by ultrafiltration; see “Materials and methods”). The loaded sample volumes were adjusted to allow for direct comparison of protein purity and assessment of protein loss. Abundant bands are marked at the right: contaminants (c), griffithsin (G), griffithsin dimer (dG). The ‘pH 8’ purification was performed in QIAGEN 6xHis-tag purification buffer supplemented with 10 mM imidazole for extraction, 20 mM imidazole for washing and 250 mM imidazole for elution (E). The ‘pH 4’ purification was performed in an extraction buffer (buffer A in **a**) supplemented with 10 mM imidazole. Washing and elution were done in buffer C (cf. **a**), supplemented with 20 mM imidazole for washing and 250 mM imidazole for elution. The ‘pH 4, MgCl_2_’ purification is similar to the ‘pH 4’ purification, but includes 100 mM MgCl_2_ in all buffers. Eluates were dialyzed and concentrated (C) in PBS buffer. Note that pH 4 extraction effectively reduced contaminations, and yielded 360 µg soluble griffithsin per gram leaf material. Extraction with ‘pH 4, MgCl_2_’ yielded slightly more griffithsin with a similar purity, but slightly lower amounts of griffithsin were recovered after ultrafiltration

If strong enrichment by heat treatment of crude protein extracts of transplastomic plants can be achieved, the high levels of griffithsin accumulating in our transplastomic tobacco plants should result in the protein becoming readily detectable in Coomassie-stained polyacrylamide (PAA) gels. Indeed, heating of leaf protein extracts to 70 °C resulted in loss of nearly all abundant cellular proteins and retention of a single prominent band that corresponds in size to griffithsin (Fig. 4a). Testing of a number of different extraction buffers revealed that buffer composition also had an impact on the efficiency of pre-purification and the abundance and pattern of residual contaminants (Fig. 4a).

We then attempted purification taking advantage of the His-tag that had been included in the griffithsin expression construct. Based on the strong enrichment that had been obtained by sample heating (Fig. 4a), further purification to apparent homogeneity by nickel affinity purification was readily achieved (Fig. 4b), providing suitable material for activity tests and yielding approximately 360 µg pure griffithsin per gram fresh weight.

### Anti-HIV activity of chloroplast-expressed griffithsin

**Fig. 5:**
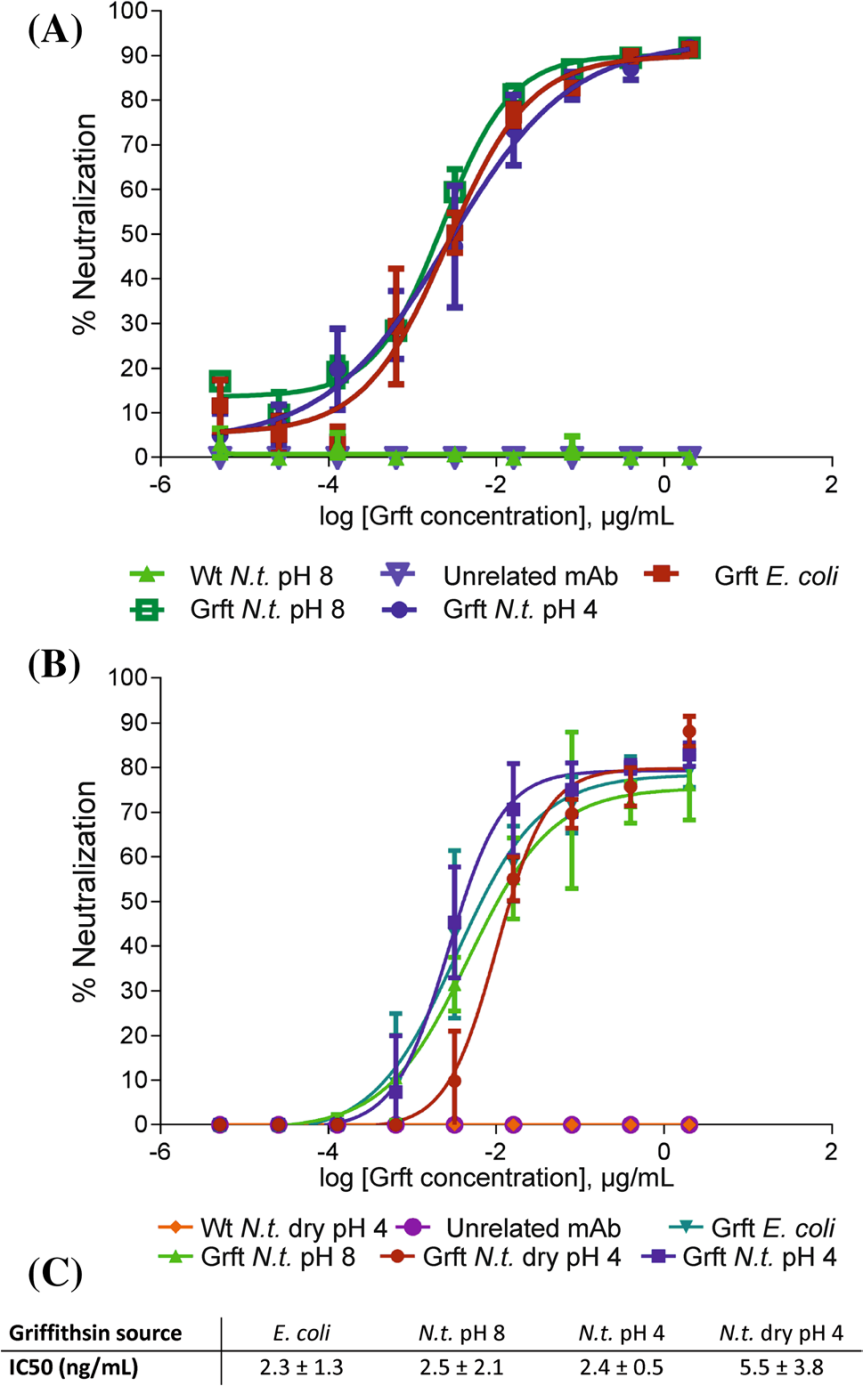
HIV neutralization assays to determine the biological activity of chloroplast-produced griffithsin. **a** HIV neutralization was compared between recombinant griffithsin produced in *E. coli* (Grft *E. coli*), wild-type tobacco protein extracts (Wt *N.t*. pH 8) and griffithsin purified from transplastomic tobacco with either His-tag purification buffer (Grft *N.t*. pH 8) or with ‘pH 4’ buffer (Grft *N.t*. pH 4; cf. Fig. 4). As an additional negative control, an anti-rabies monoclonal antibody (‘unrelated mAb’) was used. Error bars indicate the SD from three technical replicates for each experiment. **b** HIV neutralization assay comparing griffithsin extracted from freshly frozen tobacco leaves with griffithsin extracted from dried tobacco. As an additional control, extracts from dried wild-type tobacco leaves (Wt *N.t*. dry pH 4) were also included. **c** IC50 values for the different extracts representing three IC50 measurements from three experimental repeats of the HIV neutralization assay with griffithsin purified from *E. coli* and frozen tobacco leaves, and five IC50 measurements from two experimental repeats for griffithsin purified from dried tobacco leaves

To determine whether griffithsin extracted from transplastomic tobacco plants is biologically active and can prevent entry of HIV-1 into human cells, virus neutralization tests were performed (Wei et al. 2003; Montefiori 2005). To this end, cultured HeLa cells containing a HIV-inducible luciferase gene were incubated with viral particles in the presence of different concentrations of griffithsin purified from tobacco leaves. These assays revealed efficient virus neutralization by the chloroplast-produced griffithsin that was very similar to the activity of recombinant griffithsin purified from *E. coli* (Fig. 5a). These data indicate that transplastomic expression of griffithsin results in large amounts of protein that is fully active and displays high antiviral activity.

### Purification of active griffithsin from dried tobacco

**Fig. 6:**
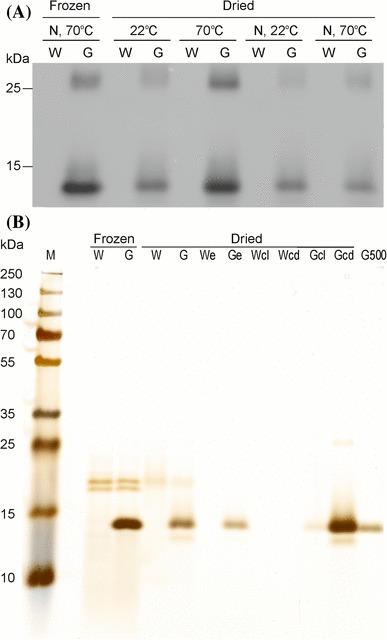
Extraction of griffithsin from dried tobacco leaves. **a** Western blot analysis of griffithsin accumulation in dried leaves. TSP was extracted from frozen or dried leaves of griffithsin-producing transplastomic (G) and wild-type plants (W). Leaves that had been dried and stored for 4 months were compared to leaves that had been frozen and stored at − 80 °C until use. 200 µL buffer were added per 100 mg ground frozen leaf material. To compensate for water loss, 29 µL/mg buffer were added to ground dried leaf material. All extractions were performed with ‘pH 4’ buffer with an incubation step at either 22 or 70 °C for 15 min. The effect of freezing the dried tobacco samples in liquid nitrogen before grinding (N) was also tested. Griffithsin and its dimer were immunodetected with a griffithsin-specific antibody. **b** Silver-stained SDS-PAA gel (10%) illustrating the purification of griffithsin from dried leaves. Extraction was performed as in **a**. The eluate (e) was ultrafiltrated resulting in a color gradient of an upper light-colored fraction (cl) and a more dark-colored lower fraction (cd). 500 ng of 6xHis-tagged griffithsin purified from *E. coli* was loaded as a reference (G500). The loaded eluate volumes were adjusted to allow for quantitative comparison of input and eluate

To facilitate rapid scale-up of the production of plant-based biopharmaceuticals, it is desirable to be able to store the raw material (plant biomass) for a longer period of time. This removes the need to respond to increasing demands by producing large amounts of plant biomass, which represents the most time-consuming step in the production process. The long-term storage procedure used in the tobacco industry relies on drying of the harvested leafy biomass. We, therefore, wanted to test whether chloroplast-synthesized griffithsin would remain stable upon drying and during storage, and retain its biological activity. We, therefore, dried leaves harvested from transplastomic griffithsin-producing plants and stored them at room temperature for up to 10 months after harvest. Afterwards, proteins were extracted and purification of griffithsin was attempted. Immunoblot analysis of total soluble protein extracts revealed high recovery of full-length griffithsin from dried tobacco and no evidence of appreciable protein degradation during drying or long-term storage (Fig. 6a).

Purification of griffithsin from dried tobacco was slightly less efficient than protein purification from fresh or frozen leaf material (Fig. 6b). Importantly, griffithsin purified from dried tobacco retained high anti-HIV activity in virus neutralization assays (Fig. 5b, c), demonstrating that dried leaf biomass facilitates long-term storage and provides suitable source material for extraction of griffithsin upon demand.

## Discussion

In the course of this work, we have investigated the possibility to produce the potent anti-HIV protein griffithsin in tobacco chloroplasts. Previous attempts to synthesize cyanovirin-N, a longer known but less potent anti-HIV protein, in trangenic chloroplasts had met with limited success (Elghabi et al. 2011). Cyanovirin-N turned out to be rather unstable in chloroplasts and, although protein accumulation could be improved by fusion to N- and/or C-terminal sequences taken from highly stable proteins, accumulation levels remained relatively low (Elghabi et al. 2011). By contrast, griffithsin, a protein of similar size but unrelated in primary amino acid sequence, accumulated in transplastomic tobacco plants to levels of up to 5% of TSP (Fig. 3). Considering the small size of the recombinant protein (of only 14.5 kDa), this is an exceptionally high expression level that suggests that, unlike cyanovirin-N, griffithsin is highly stable in transgenic chloroplasts. It is important to note that the same expression elements were used for the transplastomic expression of cyanovirin-N and griffithsin, suggesting that the strong difference in protein accumulation is due to differences in protein stability. Why griffithsin is so much more stable than cyanovirin-N is currently unclear. Although some determinants of chloroplast protein (in)stability have recently been uncovered (Apel et al. 2010; De Marchis et al. 2012; Rowland et al. 2015), we are still far from being able to explain why certain proteins are less stable than others (let alone to predict the stability of foreign proteins to be expressed in plastids).

Chloroplast-produced griffithsin could be easily purified to apparent homogeneity. The purified protein displayed high HIV-neutralizing activity and showed no evidence of cytotoxicity in bioassays with HeLa cells (Fig. 5), suggesting that transplastomic plants can provide a cost-effective and easily scalable production platform for this important antiviral agent. In the absence of an AIDS vaccine, microbicides that prevent new viral infections are likely to become an important part of future anti-HIV strategies (and perhaps, also of strategies to control other viruses that griffithsin binds to; O’Keefe et al. 2010; Barton et al. 2014).

In this work, we also explored the possibility to use dry tobacco as storable material for recombinant protein purification on demand. Interestingly, griffithsin remained stable during drying and long-term storage and retained high antiviral activity (Figs. 5, 6). The IC50 value of griffithsin extracted from dry tobacco is slightly higher than that of griffithsin purified from fresh biomass or from *E. coli* (Fig. 5c). The reason for this difference is currently unknown and could be related to some activity loss upon drying and/or storage or, alternatively, lower purity of the protein samples due to the different starting material and/or differences in sample preparation (leading to an overestimation of the griffithsin concentration and thus giving lower apparent activity). Nonetheless, similar to rice grains (Vamvaka et al. 2016), dried tobacco leaves provide an attractive option for long-term storage of large amounts of raw material for griffithsin extraction upon demand. This is of particular importance to the potential use of griffithsin in the developing world, where the demand for anti-HIV microbicides is greatest. Although griffithsin can be expressed also to high levels by transient transformation of fresh biomass (O’Keefe et al. 2009), the procedures involved are too complicated and demanding to be implemented locally.

In summary, our work reported here demonstrates the feasibility of using transplastomic plants as highly efficient and cost-effective production platform for griffithsin, the currently most promising anti-HIV agent. Moreover, we have shown that dried tobacco can serve as source material for purification of griffithsin, thus enabling quick scale-up of production and rapid response to increasing demands.

## Electronic supplementary material

Below is the link to the electronic supplementary material.

Supplementary material 1 (PDF 77 KB)
