# Supplementary material for: High-level expression of the HIV entry inhibitor griffithsin from the plastid genome and retention of biological activity in dried tobacco leaves
Source: Plant Mol Biol. 2018 Jun 9;97(4):357–70. doi: 10.1007/s11103-018-0744-7 (PMC6061503; doi:10.1007/s11103-018-0744-7)
Supplement: Supplementary file 1 — Supplementary material 1 (PDF 77 KB) [file 11103_2018_744_MOESM1_ESM.pdf]

SUPPLEMENTARY MATERIALS

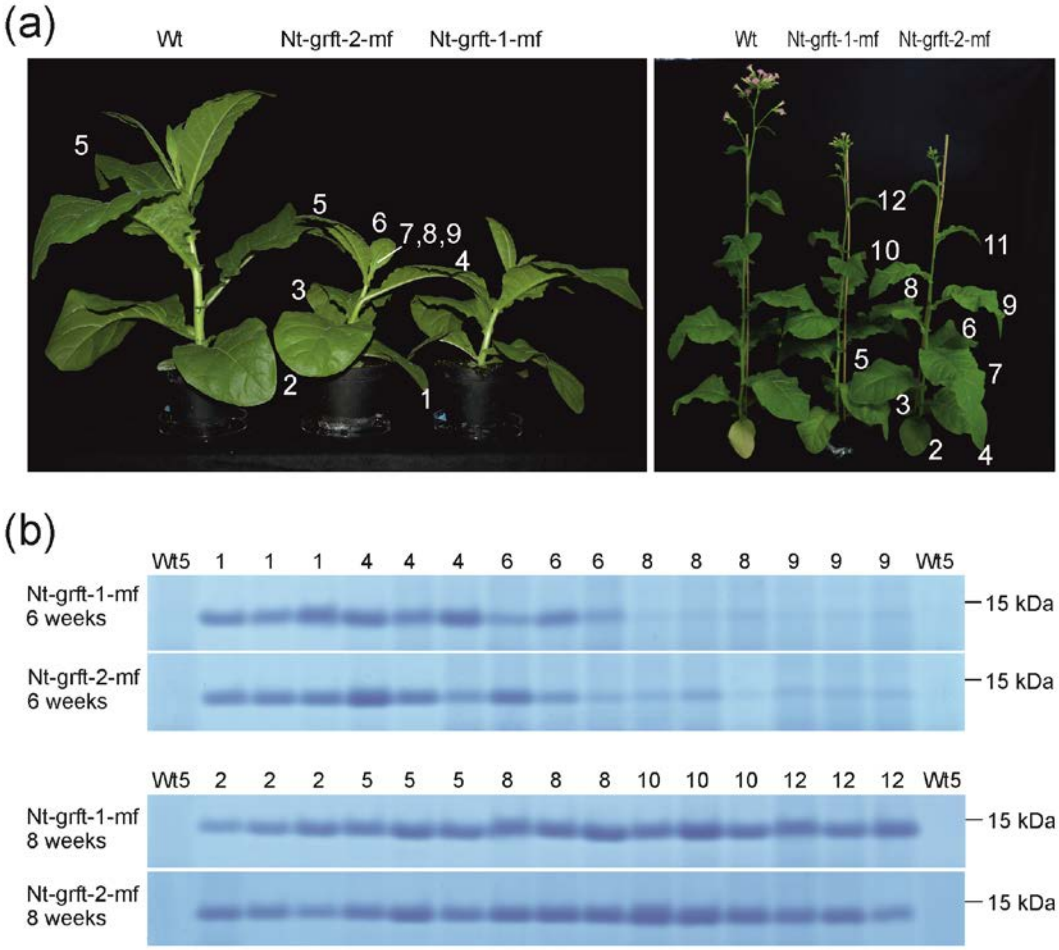

Figure S1. Accumulation of griffithsin in leaves of different ages. (**a**) Plant material and numbering of leaf samples investigated. Two marker-free transplastomic lines and a wild-type control were used to extract proteins from leaves of different ages and developmental stages and assess griffithsin accumulation. (**b**) Proteins (TSP) were extracted with the 'pH 4, $MgCl_2$' buffer (cf. Figure 4), electrophoretically separated in 10% Tris-Tricine SDS polyacrylamide gels and subsequently stained with Coomassie. For 6-week old plants, the unopened apical bud was named 'Leaf 9' and the youngest leaf distinguishable from this bud was named 'Leaf 8'. While this very young tissue

shows low griffithsin accumulation, all other leaves show strong and stable accumulation of griffithsin (that does not change substantially with leaf age). Leaf 5 of a wild-type plant (Wt5) served as negative control. Three plants per time point were analyzed.
